# Catechol-O-methyltransferase polymorphism is associated with the cortico-cerebellar functional connectivity of executive function in children with attention-deficit/hyperactivity disorder

**DOI:** 10.1038/s41598-017-04579-8

**Published:** 2017-07-07

**Authors:** Yoshifumi Mizuno, Minyoung Jung, Takashi X. Fujisawa, Shinichiro Takiguchi, Koji Shimada, Daisuke N. Saito, Hirotaka Kosaka, Akemi Tomoda

**Affiliations:** 1 Department of Child and Adolescent Psychological Medicine, University of Fukui Hospital, 23-3 Matsuokashimoaizuki, Eiheiji-cho, Yoshida-gun, Fukui, 910-1193 Japan; 2 Division of Developmental Higher Brain Functions, United Graduate School of Child Development, University of Fukui, 23-3 Matsuokashimoaizuki, Eiheiji-cho, Yoshida-gun, Fukui, 910-1193 Japan; 3 Department of Psychiatry, Harvard Medical School, Harvard University, Bldg. 120, 1st Ave., Charlestown, MA 02129 USA; 4 Research Center for Child Mental Development, University of Fukui, 23-3 Matsuokashimoaizuki, Eiheiji-cho, Yoshida-gun, Fukui, 910-1193 Japan; 5 Research Center for Child Mental Development, Kanazawa University, Kanazawa, 13-1 Takaramachi, Kanazawa-shi, Ishikawa, 920-8640 Japan

## Abstract

The cerebellum, although traditionally considered a motor structure, has been increasingly recognized to play a role in regulating executive function, the dysfunction of which is a factor in attention-deficit/hyperactivity disorder (ADHD). Additionally, catechol-O-methyltransferase (COMT) polymorphism has been reported to be associated with executive function. We examined whether the cortico-cerebellar executive function network is altered in children with ADHD and whether COMT polymorphism is associated with the altered network. Thirty-one children with ADHD and thirty age- and IQ-matched typically developing (TD) controls underwent resting-state functional MRI, and functional connectivity of executive function-related Crus I/II in the cerebellum was analysed. COMT Val158Met genotype data were also obtained from children with ADHD. Relative to TD controls, children with ADHD showed significantly lower functional connectivity of the right Crus I/II with the left dorsolateral prefrontal cortex. Additionally, the functional connectivity of children with ADHD was modulated by COMT polymorphism, with Met-carriers exhibiting significantly lower functional connectivity than the Val/Val genotype. These results suggest the existence of variations, such as ethnic differences, in COMT genetic effects on the cortico-cerebellar executive function network. These variations contribute to heterogeneity in ADHD. Further neuroimaging genetics study might lead to the development of fundamental therapies that target ADHD pathophysiology.

## Introduction

Attention-deficit/hyperactivity disorder (ADHD) is one of the most commonly diagnosed neurodevelopmental disorders in childhood, characterized by symptoms of age-inappropriate inattention, hyperactivity, and impulsivity^1^, and is clinically heterogeneous. It was recently reported that the prevalence of ADHD is approximately 7.2%^2^. ADHD is more common in boys than girls (male to female ratio is about 3:1)^3^. Children with ADHD have difficulties in daily life, and in adolescence, they often have a variety of comorbidities such as oppositional defiant disorder, conduct disorder, depression, and anxiety disorder. Additionally, ADHD often persist into adulthood, and adults with ADHD have a high risk of substance abuse disorders, suicide, and personality disorders^1^. Thus, ADHD is associated with distress and morbidity across the life span.

Impaired executive function is one of the most widespread findings in ADHD. Executive function comprises a set of neurocognitive processes that maintain an appropriate problem-solving set to attain a future goal, and involves response inhibition, working memory, and planning^4^. It is well known that the prefrontal cortex plays an important role in executive function, and that patients with ADHD often have problems in the frontal-striatal network, which includes the prefrontal cortex^5, 6^.

Although the cerebellum is traditionally considered a motor structure, it has been recognized to play a role in regulating cognitive function, including executive function^7–9^. Previous fMRI studies have revealed decreased cerebellar activation in patients with ADHD during working memory and go/no-go tasks^10–12^. Structural neuroimaging studies have revealed reduced cerebellar volumes in patients with ADHD^13–15^. Although these neuroimaging modalities focus primarily on the properties of discrete brain regions, several previous psychopathological studies have adopted a circuit perspective, which posits that psychological disorders do not stem from anomalies in discrete brain regions^16^.

In contrast, the recent resting state fMRI (rs-fMRI) approach facilitates the evaluation of neural circuit-level brain function more easily. Further, this method is suitable for young participants, and particularly for clinical populations, such as individuals with ADHD who have behavioural and compliance issues. This is because rs-fMRI has low cognitive demands (i.e., it is task-free), and the scan time is relatively short compared to task-based fMRI^17^.

The cerebellum is reported to form neural circuits with the cerebral cortex. In fact, most of the human cerebellum is linked to cerebral association networks^7^. The cerebellum is divided into sub-regions. Among these sub-regions, Crus I/II, is linked both functionally and anatomically with the prefrontal cortex, is involved in executive function^18, 19^, and is included in the executive control network^9, 20, 21^. We focused on this network, and hypothesized that the cortico-cerebellar functional connectivity of Crus I/II with the prefrontal cortex is altered in children with ADHD.

Children with ADHD have variations in clinical symptoms, even within the same diagnostic category subtype. Hence, they might have uncertain etiological and neuropsychological heterogeneity^22, 23^. Regarding disease heterogeneity, catechol-O-methyltransferase (COMT) polymorphism has been reported to be involved with the pathogenesis of ADHD, especially with respect to executive function^24, 25^. COMT is a key enzyme in the elimination of dopamine, and a single nucleotide polymorphism (SNP) Val158Met (rs4680) affects COMT activity. Those with Val158 alleles have increased COMT activity, which leads to lower dopamine levels and relatively lower prefrontal cognitive function than in individuals with Met158 alleles^26^. Interestingly, considerable COMT protein and mRNA have been detected in the cerebellum and in the frontal lobes^27^. Additionally, the cerebellum has dopaminergic innervation and dopamine receptors, similar to the prefrontal cortex^28^. Crus I/II in the cerebellum makes a circuit with the prefrontal cortex^18, 19^ such that the stimulation of the cerebellar cortex increases dopamine efflux in the prefrontal cortex^29^. Furthermore, COMT genotype is reported to affect perfusion of the frontal and lateral cerebellar regions in children during rest^30^. We therefore considered the possibility that COMT polymorphism affects cortico-cerebellar functional connectivity of executive function via changes in dopamine levels. We also hypothesized that functional connectivity affects executive function and ADHD symptoms, and leads to heterogeneity in ADHD.

The present study aimed to determine whether cortico-cerebellar functional connectivity of Crus I/II, which is implicated in executive function, is altered in children with ADHD. Further, we examined whether COMT polymorphism is associated with altered cortico-cerebellar functional connectivity of Crus I/II in children with ADHD.

## Results

### Demographic and clinical characteristics

Demographic and clinical characteristics of the study participants are presented in Table 1. Two children with ADHD were removed from the analysis owing to excessive head motion during scanning. Consequently, analyses involved data from 61 subjects, comprising 31 boys with ADHD (mean age, 9.7 years; standard deviation (SD), 2.0 years) and 30 typically developing (TD) controls (mean age, 10.6 years; SD, 2.2 years). Participants were right-handed, except for 5 children with ADHD and 2 TD controls. Twenty-four children with ADHD were combined type and 7 were predominantly inattentive type; 3 ADHD patients had oppositional defiant disorder as comorbid disorders. Thirteen children with ADHD were medication naïve. There were no significant differences in age, handedness, socioeconomic status (SES), full scale intelligence quotient (FSIQ), or head motion parameters such as mean frame-to-frame root mean squared (RMS) motion and frame-wise displacement (FD) between the both groups. However, there were significant differences in inattention, hyperactivity-impulsivity, and executive function scores as evaluated by the Conners 3rd Edition (Conners 3) between groups (all *p* < 0.001; Table 1).

**Table 1:** Demographic data of the participants.

	TD	ADHD			Difference (p value)	
		ALL	COMT genotype		TD-ADHD	COMT genotype
			Met-carriers	Val/Val		Met-carriers-Val/Val
Subjects (n)	30	31	16	15	—	—
Age (years)	10.6 (2.2)	9.7 (2.0)	9.9 (1.9)	9.4 (2.0)	0.080	0.454
Gender (n, male)	30	31	16	15	—	—
Handedness (n, R/L)	28/2	26/5	13/3	13/2	0.246	0.682
SES	31.0 (17.2)	37.9 (12.1)	37.1 (14.0)	38.7 (10.0)	0.077	0.716
FSIQ	103.8 (10.2)	97.4 (14.8)	99.1 (16.4)	95.5 (13.3)	0.056	0.515
Conners IN (T)	45.4 (7.9)	71.4 (13.2)	68.4 (12.4)	74.5 (13.8)	<0.001	0.207
Conners HY (T)	42.8 (4.1)	67.1 (14.4)	64.6 (13.3)	69.9 (15.5)	<0.001	0.311
Conners EF (T)	45.3 (5.7)	65.7 (9.5)	66.5 (9.0)	68.1 (10.3)	<0.001	0.657
RMS mean displacement (mm)	0.04 (0.03)	0.05 (0.03)	0.04 (0.02)	0.05 (0.03)	0.533	0.132
Mean FD (mm)	0.17 (0.06)	0.19 (0.07)	0.19 (0.08)	0.19 (0.06)	0.137	0.894

ADHD, attention-deficit/hyperactivity disorder; TD, typically developing; COMT, catechol-O-methyltransferase; SES, socioeconomic status; FSIQ, full scale intelligence quotient; IN, inattention; HY, hyperactivity/impulsivity; EF, executive function; T, T-score; RMS, root mean square; FD, frame-wise displacement.

Based on the COMT genotype, children with ADHD were divided into Val/Val (n = 15) and Met-carriers (Val/Met: n = 13, Met/Met: n = 3). No significant deviation from Hardy-Weinberg equilibrium was observed for the SNP in this study. There were no significant differences between Met-carriers and Val/Val groups on all demographic data including Conners scores (inattention (T): *p* = 0.207, hyperactivity/impulsivity (T): *p* = 0.311, executive function (T): *p* = 0.657).

### Seed-based functional connectivity

**Figure 1:**
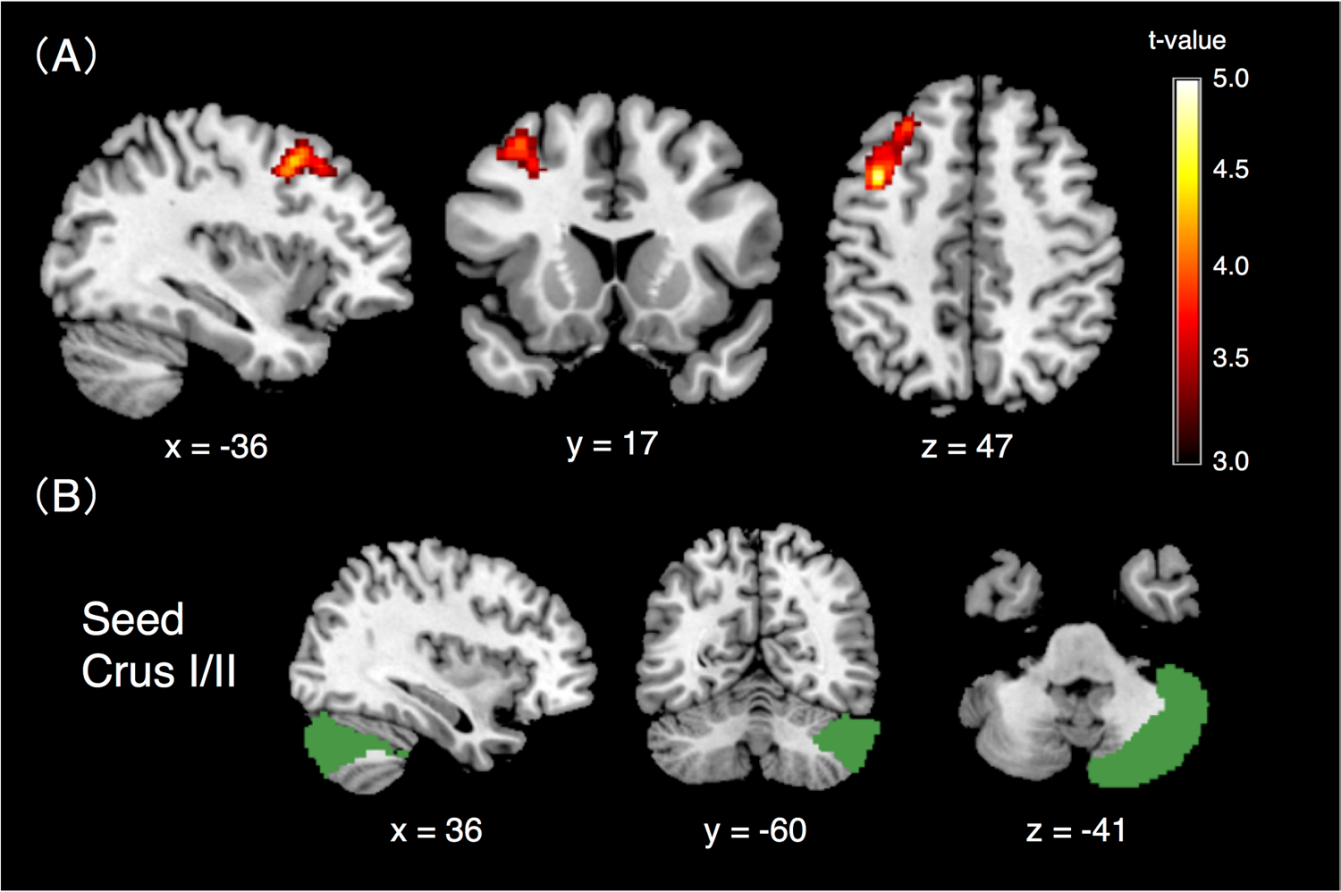
(**A**) Significant difference in functional connectivity of Crus I/II between ADHD and TD groups by seed-based analysis. Children with ADHD showed significantly lower functional connectivity of the right Crus I/II with the left DLPFC (MNI coordinates, *x* = −38, *y* = 10, *z* = 48; cluster size = 311 voxels; *p* = 0.011, FWE corrected at cluster level). (**B**) Crus I/II, which was used as seed (Figure shows only right Crus I/II). TD, typically developing; DLPFC, dorsolateral prefrontal cortex; MNI, Montreal Neurological Institute; FWE, family wise error.

Compared to TD controls, children with ADHD showed significantly lower functional connectivity of the right Crus I/II with the left dorsolateral prefrontal cortex (DLPFC; Montreal Neurological Institute (MNI) coordinates: *x* = −38, *y* = 10, *z* = 48; cluster size = 311 voxels; *p* = 0.011, family wise error (FWE) corrected at cluster level). No regions were significantly associated with higher functional connectivity in children with ADHD (Fig. 1). Significant correlations were found between functional connectivity of the right Crus I/II with the left DLPFC and Conners executive function scores (*r* = −0.447, *p* < 0.001) across all subjects, although no significant correlations were found within ADHD or TD groups (ADHD, *r* = −0.142, *p* = 0.447; TD, *r* = 0.041, *p* = 0.830).

### Genotype differences in ADHD-related functional connectivity of Crus I/II

**Figure 2:**
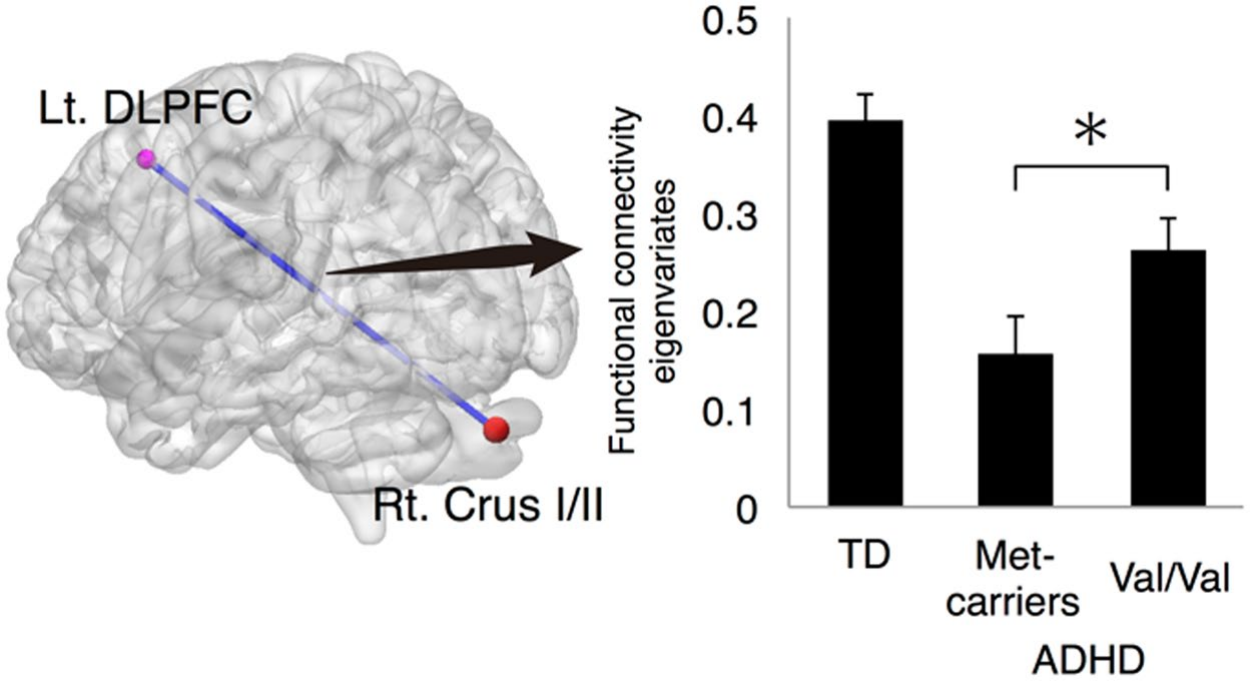
Functional connectivity of the right Crus I/II with the left DLPFC in three groups (TD, ADHD Met-carriers, and ADHD Val-homozygotes). Lt, left; Rt, right; TD, typically developing; DLPFC, dorsolateral prefrontal cortex; ^﻿*^ *p* < 0.05.﻿

Finally, we examined if COMT polymorphism was associated with the ADHD-related altered functional connectivity between the right Crus I/II and left DLPFC. The functional connectivity eigenvariates from the identified cluster were extracted and used for between-genotype comparisons. Met-carriers exhibited significantly lower functional connectivity of the right Crus I/II with the left DLPFC than the Val/Val genotype (*p* = 0.021; Fig. 2), suggesting that functional connectivity in the ADHD group was modulated by COMT polymorphism.

## Discussion

The present study elucidated whether the cortico-cerebellar network involved with executive function is abnormal in children with ADHD, by assessing functional connectivity of the bilateral Crus I/II in the cerebellum, and whether COMT polymorphism is associated with the network. This study provides preliminary evidence of lower functional connectivity of the right Crus I/II in the cerebellum with the left DLPFC in children with ADHD versus TD controls. Further, we found that the lower functional connectivity of children with ADHD was modulated by COMT polymorphism, with Met-carriers exhibiting significantly lower functional connectivity than the Val/Val genotype. These results suggest the possibility of gene-brain interactions between the cortico-cerebellar executive function network and COMT polymorphism, which influence heterogeneity in ADHD.

DLPFC plays an important role in regulating executive function, including working memory and attention^31^. Previous fMRI studies using attention tasks in children with ADHD specifically reported functional abnormalities of the DLPFC^32, 33^. Several rs-fMRI studies have reported abnormal fronto-cerebellar networks in children with ADHD^34–36^. Interestingly, Li *et al*.^35^ reported reduced functional connectivity between the left orbitofrontal cortex and left lobule VI in the cerebellum in children with ADHD. This finding is consistent with that of our study, by suggesting that connectivity of the fronto-cerebellar network is decreased in ADHD. Lobule VI in the cerebellum primarily contributes to the salience network^9^, which plays a crucial role in identifying the most biologically and cognitively relevant events, so as to adaptively guide attention and behaviour; the region has been suggested as being related to ADHD^37^. In contrast, in the present study we focused particularly on Crus I/II, which subserves executive function. A meta-analysis of voxel-based morphometric studies reported decreased volume of the right Crus I in children with ADHD^13^. Moreover, it has been reported that inattention and hyperactivity/impulsivity symptoms are caused by damage to the cerebellar posterior lobe, which includes Crus I/II^38^. Previous rs-fMRI studies reported that both Crus I/II and DLPFC are included in the executive control network^9, 20, 21^. Together, these results suggest that reduced functional connectivity of the Crus I/II with the DLPFC in children with ADHD affects executive function, which may lead to ADHD symptoms.

Curiously, we found that COMT polymorphism was associated with functional connectivity of the right Crus I/II with the left DLPFC in children with ADHD, and the functional connectivity in Met-carriers was significantly lower than in Val/Val individuals. Generally, those with Val158 alleles have increased COMT activity, leading to lower dopamine levels, which in turn leads to deficits in prefrontal cognitive function when compared to individuals with Met158 alleles^26^. It has been also reported that COMT gene variants have particular evolutionary advantages. Specifically, Val158 alleles may be associated with an advantage in the processing of aversive stimuli, while Met158 alleles may be associated with an advantage in memory and attention tasks^39^. In the present study, Met-carriers had lower ADHD-related functional connectivity than Val/Val individuals had and TD controls, showing that Japanese children with ADHD exhibit the opposite pattern to that found in Caucasians. Our recent study and those of others have demonstrated that COMT may have differential effects across ethnic samples^40, 41^. While Caucasians with the Met-allele have better cognitive performance than Val homozygotes^42, 43^, Asians with Val homozygotes perform better in cognitive tests than those with the Met-allele^44, 45^. Similarly, Met-carrier individuals with ADHD have less striatal gray matter volume than those with the Val/Val genotype in Japanese samples in contrast to Caucasians^40, 41, 46^. Thus, the present study supports the existence of an ethnic difference in the COMT genetic concomitants between Caucasians and Asians. Such ethnic differences in COMT genetic effects may be explained by the theoretical inverted-U model. This model describes an association between dopamine levels and cognitive performance dependent on the COMT Val158Met genotype. According to this inverted-U model^39, 47, 48^, optimal cognitive performance is not associated with maximal, but rather optimal dopamine levels. It can be proposed that baseline dopamine levels on the curve are influenced by an interaction between ethnicity and COMT genotype. Caucasian individuals fall on the left side of the inverted U-shaped curve. Therefore, COMT Val homozygosity is more strongly associated with poorer cognitive performance in these individuals. In contrast, Asian individuals fall on the right side of this curve. As a result, COMT Met carriers who are Asian have poorer cognitive function. High COMT protein and mRNA levels have been detected in the cerebellum and in the frontal lobes^27^. It is also known that stimulation of the cerebellar cortex increases dopamine efflux in the prefrontal cortex. Together, these observations suggest that COMT polymorphism may affect dopamine levels, which may then affect the cortico-cerebellar executive function network. Furthermore, COMT may have different effects on ADHD-related functional connectivity in different ethnic groups and on cognitive performance in ADHD. This gene-brain interaction between the cortico-cerebellar executive function network and COMT polymorphism may be associated with heterogeneity in ADHD. However, although we have discussed this finding in terms of a potential cause and effect mechanism, it must be emphasized that our evidence only supports an association.

Although the DSM criteria are categorical, being primarily based on the signs and symptoms present, in fact categorical diagnosis remains uncertain with respect to disease heterogeneity. Thus, studies that adopt this categorical classification are limited, in that a categorical diagnosis does not necessarily capture currently unknown mechanisms of dysfunction. The National Institute of Mental Health in the United States launched the Research Domain Criteria (RDoC) project to create a framework for research on pathophysiology, especially for genomics and neuroscience. They expect that identifying syndromes on the basis of pathophysiology will eventually improve outcomes for psychiatric diseases, as well as cancer, heart disease, and infectious diseases^22^. Regarding capturing the underlying pathophysiological mechanisms, brain-imaging genetics studies, which primarily identify genes that influence variation among brains, are increasingly prevalent. *In vivo* neuroimaging studies that elucidate endophenotypes of neurodevelopmental disorders combined with genetic actions will help to identify the risk genes, and investigations of these brain function related genes will help validate their association with disease^49^. Although some imaging genetics studies of ADHD have been reported^50, 51^, the present study is the first to examine the influence of COMT polymorphism on resting-state functional connectivity in patients with ADHD. Neurodevelopmental disorders such as ADHD are addressed as disorders of brain circuitry^16, 22^. Recently, nodal centrality measures (e.g., betweenness, centrality, and eigenvector centrality) have been used to quantify brain network connectivity. Imaging genetics studies using these analyses would help to clarify the underlying neuronal mechanisms and provide a new framework for classification based on such mechanisms. This could lead to the development of fundamental treatment strategies that consider the underlying pathophysiology.

This study is subject to several limitations. First, the children with ADHD included patients of combined type or predominantly inattentive type, and patients who were not drug naïve. Medication status might affect functional connectivity^52^. Second, gene-gene interactions were not considered. The COMT genetic effects may be influenced by other common variations in dopamine-regulating genes, such as monoamine oxidase^41^. We also did not examine the genotypes of TD controls, and the effects of COMT polymorphism in TD controls. In the present study, we focused on heterogeneity of ADHD as it related to COMT polymorphism. Third, dysfunction of the DLPFC has been also described in developmental coordination disorder (DCD)^53^, which is frequently comorbid with ADHD. We evaluated 11 of 31 patients with ADHD in the present study using a standard assessment tool for DCD, the Movement Assessment Battery for Children Second Edition (M-ABC2), which was recently reported in another study^54^. Although 10 patients had no difficulty with movement, one was at risk of having movement difficulties. Additionally, we did not evaluate the remaining patients with ADHD using the M-ABC2. Hence, some patients with ADHD in the present study may also have had DCD, which may have affected the results. Finally, participants were of a wide range of ages and the sample size of the patient group was relatively small. We must consider that the brain changes dynamically with age^14, 55^, although we matched ages between groups and included age as a covariate to minimize its effect on the results. Our observations have been made in small samples and are thus speculative and need to be replicated in larger samples. Further, although rs-fMRI studies are increasingly used to understand the effects of disease processes on neuronal networks, caution is required when interpreting functional connectivity changes in cortico-cerebellar networks.

In conclusion, this study showed that children with ADHD had significantly lower functional connectivity between the right Crus I/II and the left DLPFC compared to TD controls, and COMT polymorphism was associated with functional connectivity in children with ADHD. These results suggest that gene-brain interactions between cortico-cerebellar executive function networks and COMT polymorphism influence heterogeneity in ADHD. Further imaging genetics studies using rs-fMRI might clarify the underlying pathophysiological mechanisms, which might lead to the development of fundamental therapies that accord with pathophysiology.

## Methods

### Participants

The protocol was approved by the Ethics Committee of the University of Fukui (Assurance no. 20110104) and was conducted in accordance with the Declaration of Helsinki and the Ethical Guidelines for Clinical Studies of the Ministry of Health, Labour and Welfare of Japan. After a complete explanation of the study, all participants and the parent(s) provided written informed consent and assent for participation in this study. This study is registered with the University Hospital Medical Information Network (UMIN000025830).

In total, 33 boys with ADHD, who were referred to our laboratory, were recruited for the present study at the University of Fukui Hospital, Japan. The diagnosis of ADHD was based on the Diagnostic and Statistical Manual of Mental Disorders, Fifth Edition (DSM-5)^1^, and comorbid diseases were evaluated by semi-structured diagnostic interview, via the Mini International Neuropsychiatric Interview for Children and Adolescents - Japanese version (MINI-KID)^56^.

Age-matched controls consisted of 30 TD school boys aged 7–14 years, who were recruited from the community. We confirmed the presence of no psychiatric diseases in TD controls by MINI-KID. This group had no family history of psychiatric diseases.

Intellectual capacities were estimated via the Wechsler Intelligence Scale for Children-Fourth (WISC-IV)^57^. Parents of each group were asked to complete Conners 3 instrument^58^ to evaluate executive function, inattention, and hyperactivity/impulsivity symptoms. The Hollingshead Index of Social Position was administered as a composite measure of SES^59^, and handedness was assessed according to the Edinburgh Handedness Inventory^60^.

Exclusion criteria for both groups were any contraindications for MRI, FSIQ < 70, any history of other neurodevelopmental disorders (e.g., autism spectrum disorder, specific learning disorder), severe head trauma, neurological abnormalities (e.g., epilepsy, tics), psychiatric diseases except for oppositional defiant disorder and conduct disorder, any history of child maltreatment, and excessive head motion (over 2.5 mm, 2.5 degree, and mean FD 0.5 mm) during the scanning. All participants were medication-free prior to MRI for at least 5 times half-lives, including methylphenidate and atomoxetine, consistent with previous studies^61^.

### Genotyping

Genomic DNA was extracted from the peripheral blood via the standard phenol-chloroform method using the QIAamp DNA Micro Kit (QIAGEN, Tokyo, Japan) in accordance with the manufacturer’s instructions. Two children with ADHD underwent extraction from oral mucosa because they rejected blood sampling. COMT Val158Met polymorphism (rs4680) was genotyped by real-time polymerase chain reaction (RT-PCR) analysis using the StepOnePlus System (Applied Biosystems, Tokyo, Japan) in accordance with standard protocols provided by the manufacturer.

### Brain imaging

Functional images were acquired with a T2*-weighted gradient-echo echo-planar imaging (EPI) sequence via a 3-T scanner (Discovery MR 750; General ElectricMedical Systems, Milwaukee, WI) and a 32-channnel head coil. In total, 201 volumes were acquired for a total scanning time of 7 minutes 42 seconds. Each volume consisted of 40 slices, with a thickness of 3.5 mm and a 0.5-mm gap to cover the entire brain. The time interval between each successive acquisition of the same slice (repetition time, TR) was 2300 ms, with an echo time (TE) of 30 ms, and a flip angle (FA) of 81°. The field of view (FOV) was 192 × 192 mm, and the matrix size was 64 × 64, yielding volume dimensions of 3 × 3 mm. The participants were instructed to stay awake but close their eyes and think of nothing in particular. Participant movement was further minimized by the placement of memory-foam pillows around their head^17^.

### fMRI data preprocessing

fMRI data were analysed using SPM12 (http://www.fil.ion.ucl.ac.uk/spm/), a data processing assistant, and resting-state fMRI software (DPARSF)^62^ with the following steps. First, the initial 10 volumes were discarded, and slice-timing correction was not applied, followed by spatial realignment of 191 volumes to the mean volume. The signal from each slice was realigned temporally to that obtained from the middle slice using sinc interpolation. To control for motion confounds in our data, we investigated the effects of head motion by computing the mean frame-to-frame RMS motion^63^, and FD^64^ obtained during the realignment process. The re-sliced volumes were normalized to MNI space with a voxel size of 2 × 2 × 2 mm^3^ using the EPI template provided by SPM12. The normalized images were spatially smoothed with a 6-mm Gaussian kernel. Next, the linear trend in the time series was removed, and temporal bandpass filtering (0.01–0.08 Hz) was performed to reduce the effects of low-frequency drift and high-frequency noise. The non-neural noise in the time series was controlled, and several sources of spurious variance (e.g., the Friston 24-parameter model, white matter signals, and cerebrospinal fluid signals) were eliminated from the data through linear regression^17^. We also performed a similar analysis after we removed frames with FD > 0.5 mm (scrubbing) during preprocessing^65^. We describe the results in the supplementary information section (Figures S1 and S2).

### Seed-based functional connectivity

We analysed seed-based functional connectivity using a seed region (region of interest, ROI) correlation approach. Bilateral Crus I/II in the cerebellum was selected as the seed region because Crus I/II is particularly involved with executive function^9, 66^, is linked to the prefrontal cortex functionally and anatomically^18, 19^, and is included in executive control network^9, 20, 21^. The ROI was isolated using an automated anatomical labelling template^67^, implemented in the WFU PickAtlas software^68^. The mean time course of all voxels in each seed was used to calculate voxel-wise linear correlations (Pearson’s correlations) for the whole brain, and individual’s *r*-values were then normalized to *z*-values using Fisher’s *z*-transformation. For group comparisons, we examined differences via a two-sample *t*-test (ADHD versus TD), including age and FSIQ as covariates in the model due to their potential confounding effects^34^. The statistical threshold was set at *p* < 0.05 with FWE correction for multiple comparisons at the cluster level (height threshold of *p* < 0.001). Further, the relationship between ADHD-related functional connectivity of Crus I/II and Conners executive function scores was investigated using Pearson’s correlation coefficients.

### Genotype differences in ADHD-related functional connectivity of Crus I/II

To further determine whether COMT polymorphism was associated with ADHD-related functional connectivity alterations of Crus I/II, the ADHD-related functional connectivity of Crus I/II was compared between the two COMT groups (Val/Val, Met-carriers), using Welch’s *t*-tests. Eigenvariates were extracted from the identified cluster. The eigenvariate is a measure of the central tendency of the cluster, which is robust to the heterogeneity of the response within the cluster. The functional connectivity eigenvariates indicate the degree of functional connectivity of Crus I/II with the cluster^69^.

### Statistical analyses

Demographic data are expressed as the mean ± SD. The clinical values were compared between groups using Welch’s *t*-test for numerical variables and chi-square tests for categorical valuables. All statistical tests (Welch’s *t*-test, chi-square test, and Pearson’s correlation coefficients) were two-tailed; *p-*values less than 0.05 were considered statistically significant. Statistical analyses were performed using the Statistical Package for the Social Sciences 23 software (SPSS, Chicago, IL).

## Electronic supplementary material

Supplementary information
