# Supplementary material for: Catechol-O-methyltransferase polymorphism is associated with the cortico-cerebellar functional connectivity of executive function in children with attention-deficit/hyperactivity disorder
Source: Sci Rep. 2017 Jul 7;7:4850. doi: 10.1038/s41598-017-04579-8 (PMC5501850; doi:10.1038/s41598-017-04579-8)
Supplement: Supplementary file 1 — Supplementary information [file 41598_2017_4579_MOESM1_ESM.pdf]

Supplementary information

Catechol-O-methyltransferase polymorphism is associated with the cortico-cerebellar functional connectivity of executive function in children with attention-deficit/hyperactivity disorder

Yoshifumi Mizuno M.D.[a, b], Minyoung Jung Ph.D.[c], Takashi X. Fujisawa Ph.D.[b, d], Shinichiro Takiguchi M.D., Ph.D.[a], Koji Shimada Ph.D.[b, d], Daisuke N. Saito Ph.D.[e], Hirotaka Kosaka M.D., Ph.D.[a, b, d], Akemi Tomoda M.D., Ph.D.[a, b, d, *]

[a]Department of Child and Adolescent Psychological Medicine, University of Fukui Hospital, 23-3 Matsuokashimoaizuki, Eiheiji-cho, Yoshida-gun, Fukui 910-1193, Japan

[b]Division of Developmental Higher Brain Functions, United Graduate School of Child Development, University of Fukui, 23-3 Matsuokashimoaizuki, Eiheiji-cho, Yoshida-gun, Fukui 910-1193, Japan

[c]Department of Psychiatry, Harvard Medical School, Harvard University, Bldg. 120, 1st Ave., Charlestown, MA 02129, USA

[d]Research Center for Child Mental Development, University of Fukui, 23-3 Matsuokashimoaizuki, Eiheiji-cho, Yoshida-gun, Fukui 910-1193, Japan

[e]Research Center for Child Mental Development, Kanazawa University, Kanazawa, 13-1 Takaramachi, Kanazawa-shi, Ishikawa, 920-8640, Japan

Supplementary information

Scrubbing

Here we described the results of our analysis after removing frames with framewise displacement > 0.5 mm (scrubbing) during preprocessing. Scrubbing did not influence the results very much (Figures S1 and S2).

Degree centrality

We performed a preliminary analysis of degree centrality using resting-state fMRI software (DPARSF) and compared it between the ADHD and TD groups. This analysis revealed no significant differences in degree centrality between the two groups.

Figure legends

Figure S1. (A) Significant difference in functional connectivity of Crus I/II between ADHD and TD groups by seed-based analysis. Children with ADHD showed significantly less functional connectivity of the right Crus I/II with the left DLPFC (MNI coordinates, x=-38, y=10, z=48; cluster size = 366 voxels; p = 0.004, FWE corrected at cluster level). (B) Crus I/II, which was used as seed (Figure shows only right Crus I/II). TD, typically developing; DLPFC, dorsolateral prefrontal cortex; MNI, Montreal Neurological Institute; FWE, family wise error.

Figure S2. Functional connectivity of the right Crus I/II with the left DLPFC in three groups (TD, ADHD Met-carriers, and ADHD Val-homozygotes). Lt, left; Rt, right; TD, typically developing; DLPFC, dorsolateral prefrontal cortex; MNI, Montreal Neurological Institute.

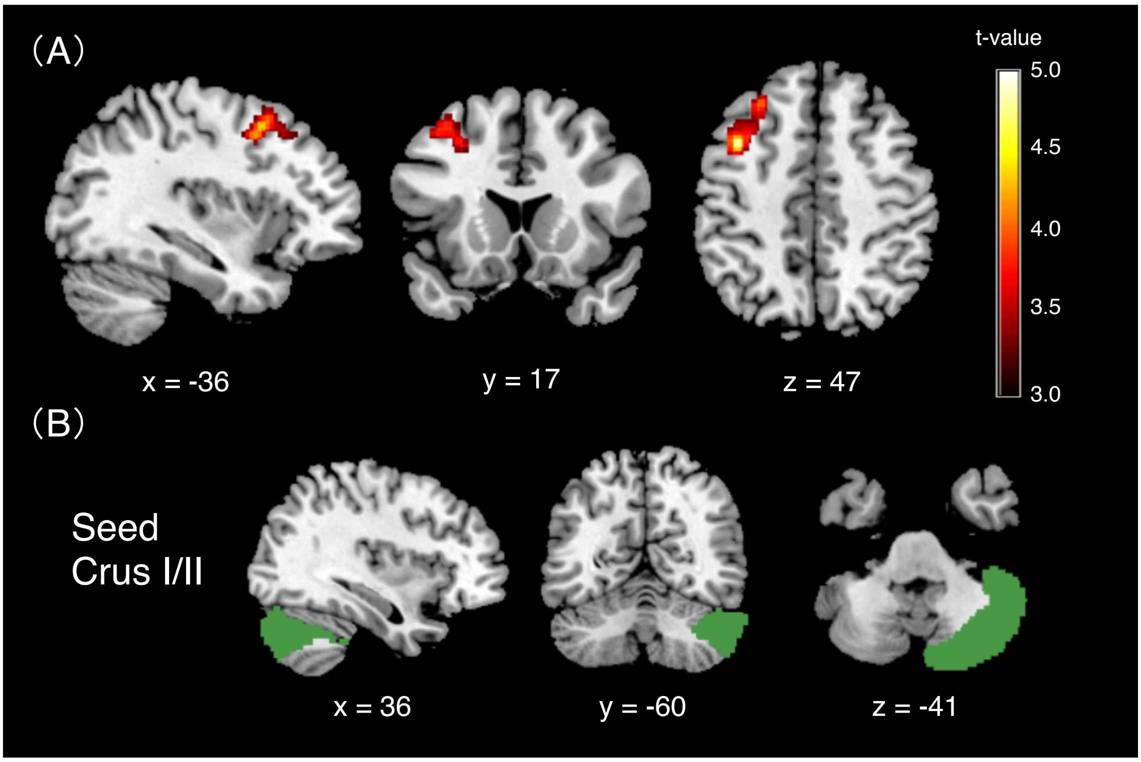

Figure S1

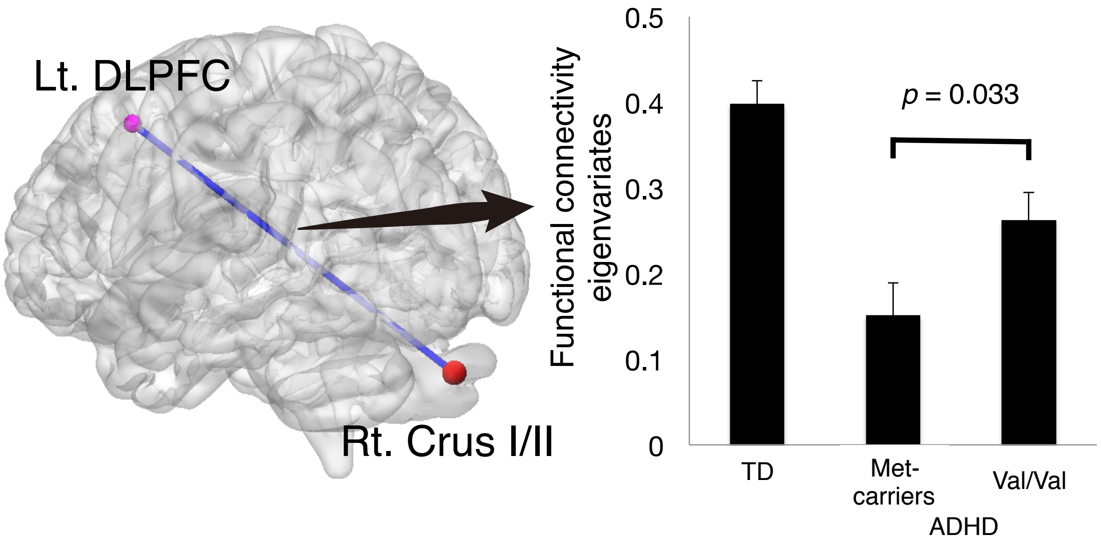

Figure S2
